# Health Care Providers as Agents of Change: Integrating PrEP With Other Sexual and Reproductive Health Services for Adolescent Girls and Young Women

**DOI:** 10.3389/frph.2021.668672

**Published:** 2021-05-28

**Authors:** Gabrielle O'Malley, Kristin M. Beima-Sofie, Stephanie D. Roche, Elzette Rousseau, Danielle Travill, Victor Omollo, Sinead Delany-Moretlwe, Linda-Gail Bekker, Elizabeth A. Bukusi, John Kinuthia, Gena Barnabee, Julie C. Dettinger, Anjuli D. Wagner, Jillian Pintye, Jennifer F. Morton, Rachel E. Johnson, Jared M. Baeten, Grace John-Stewart, Connie L. Celum

**Affiliations:** ^1^Department of Global Health, University of Washington, Seattle, WA, United States; ^2^The Desmond Tutu HIV Centre, University of Cape Town, Cape Town, South Africa; ^3^Wits Reproductive Health and HIV Institute (Wits RHI), Faculty of Health Sciences, University of Witwatersrand, Johannesburg, South Africa; ^4^Centre for Microbiology Research, Kenya Medical Research Institute, Nairobi, Kenya; ^5^Departments of Obstetrics and Gynecology, University of Washington, Seattle, WA, United States; ^6^Department of Research & Programs, Kenyatta National Hospital, Nairobi, Kenya; ^7^Department of Medicine, University of Washington, Seattle, WA, United States; ^8^Department of Epidemiology, University of Washington, Seattle, WA, United States; ^9^Department of Pediatrics, University of Washington, Seattle, WA, United States

**Keywords:** PrEP, AGYW HIV prevention, sexual and reproductive health services, service integration, health care worker motivation

## Abstract

**Background:** Successful integration of pre-exposure prophylaxis (PrEP) with existing reproductive health services will require iterative learning and adaptation. The interaction between the problem-solving required to implement new interventions and health worker motivation has been well-described in the public health literature. This study describes structural and motivational challenges faced by health care providers delivering PrEP to adolescent girls and young women (AGYW) alongside other SRH services, and the strategies used to overcome them.

**Methods:** We conducted in-depth interviews (IDIs) and focus group discussions (FGDs) with HCWs from two demonstration projects delivering PrEP to AGYW alongside other SRH services. The Prevention Options for the Women Evaluation Research (POWER) is an open label PrEP study with a focus on learning about PrEP delivery in Kenyan and South African family planning, youth mobile services, and public clinics at six facilities. PrIYA focused on PrEP delivery to AGYW via maternal and child health (MCH) and family planning (FP) clinics in Kenya across 37 facilities. IDIs and FGDs were transcribed *verbatim* and analyzed using a combination of inductive and deductive methods.

**Results:** We conducted IDIs with 36 participants and 8 FGDs with 50 participants. HCW described a dynamic process of operationalizing PrEP delivery to better respond to patient needs, including modifying patient flow, pill packaging, and counseling. HCWs believed the biggest challenge to sustained integration and scaling of PrEP for AGYW would be lack of health care worker motivation, primarily due to a misalignment of personal and professional values and expectations. HCWs frequently described concerns of PrEP provision being seen as condoning or promoting unprotected sex among young unmarried, sexually active women. Persuasive techniques used to overcome these reservations included emphasizing the social realities of HIV risk, health care worker professional identities, and vocational commitments to keeping young women healthy.

**Conclusion:** Sustained scale-up of PrEP will require HCWs to value and prioritize its incorporation into daily practice. As with the provision of other SRH services, HCWs may have moral reservations about providing PrEP to AGYW. Strategies that strengthen alignment of HCW personal values with professional goals will be important for strengthening motivation to overcome delivery challenges.

## Introduction

Adolescent girls and young women (AGYW) in sub-Saharan Africa have among the highest HIV incidence rates globally. In southern and eastern Africa, HIV prevalence among young women aged 15–24 is approximately three times as high as males in the same age group (1, 2). Although voluntary medical male circumcision, community-wide HIV testing, and increased antiretroviral therapy (ART) coverage for people living with HIV have all contributed to reductions in global HIV incidence, young African women have not significantly benefited from these prevention strategies (3, 4). HIV incidence in African AGYW has remained ~4% in recent HIV vaccine and prevention trials (5, 6). Oral tenofovir-based pre-exposure prophylaxis (PrEP) has been proven safe and effective in preventing HIV (7), and has tremendous potential to empower young women to protect themselves (8, 9). Longer-acting PrEP formulations with the dapivirine vaginal ring (10, 11) and injectable cabotegravir (12) have also been shown to be safe and efficacious and will provide a choice of PrEP options once they have received regulatory approval. Although oral tenofovir-based PrEP has been included in national guidelines in most sub-Saharan African countries, identifying and refining pathways for its delivery to AGYW is a work in progress.

Integrating PrEP with other sexual and reproductive health (SRH) services has significant potential for reaching young women (13). Over the past 20 years, advocates have argued that integrating HIV testing, care, and treatment into SRH services produces benefits and efficiencies for both facilities and clients (14–17). Some evidence has shown that such integration at antenatal care clinics (ANC), maternal and child health (MCH) clinics, and family planning (FP) clinics results in increased provision, uptake, and efficiency of services while improving client satisfaction and service outcomes (14, 16, 18, 19). However, process evaluations have shown a high degree of heterogeneity in both implementation and results (20–22).

Recently, African countries with high HIV prevalence have begun promoting the integration of PrEP into routinely delivered SRH services. Theoretically, health care workers (HCWs) in FP clinics may be especially well-positioned to counsel on PrEP as they already counsel women on SRH and routinely screen for sexual behavior and HIV risk factors (23, 24). Although there are some early indications that integrated delivery in these clinics is feasible and acceptable to clients, and reach significant numbers of women (23, 25), there is little in the peer- reviewed literature describing potential challenges to sustained, scaled implementation in routine practice settings. This study describes experiences of HCWs delivering PrEP to adolescent girls and young women (AGYW) alongside other SRH services in Kenya and South Africa, the challenges they foresee in scaling integrated PrEP and SRH services, and the strategies they suggest to overcome them.

## Materials and Methods

### Study Settings

Qualitative data were drawn from two different studies exploring PrEP delivery to AGYW aged 15–25 as an integrated component of SRH services. The Prevention Options for Women Evaluation Research (POWER) study was a prospective cohort implementation science study to evaluate PrEP delivery in six facilities across three locations: two family planning clinics in Kisumu, Kenya; a mobile clinic serving youth in disadvantaged communities and a primary care facility in Cape Town, South Africa; and an adolescent friendly clinic and a primary health care facility in Johannesburg, South Africa. The PrEP Implementation in Young Women and Adolescents (PrIYA) study evaluated the integration of PrEP delivery into existing SRH healthcare services provided in FP and MCH clinics at 37 facilities in Kisumu, Kenya (26). POWER hired study staff to integrate PrEP into other SRH services at four primary study facilities and these staff then provided technical assistance to provide PrEP at two additional facilities. PrIYA hired study nurses to integrate PrEP into existing SRH services at 16 facilities and then provided technical assistance to 21 additional facilities.

The POWER study was reviewed and approved by ethics committees at the University of Cape Town, the University of the Witwatersrand in Johannesburg, the Kenya Medical Research Institute (KEMRI), and the University of Washington. The PrIYA study was reviewed and approved by the Kenyatta National Hospital Ethics and Research Committee and the University of Washington Institutional Review Board.

### Data Collection

As part of the POWER study, we conducted in-depth interviews (IDIs) from October 2019 through January 2020 using a semi-structured interview guide. We used purposive sampling to recruit HCWs with different roles and responsibilities in PrEP delivery. Sample size was based on estimates of informational saturation and through extensive conversations with study team members who have extensive experience working with the study facilities. Interviews were conducted in English by two highly experienced, formally trained American qualitative researchers. Interviews lasted between 60 and 90 min and were audio recorded. Interviewers debriefed impressions and experiences of the interview process on telephone calls and via emails throughout the process. POWER staff in Kenya and South Africa hired individuals to transcribed the audio recordings of the interviews *verbatim* and audio recordings were reviewed and quality checked by the primary interviewers. As part of the PrIYA study, we conducted focus group discussions (FGDs) between October and December 2018. The sample size for FGDs was based on estimates of information saturation and arrived at through extensive discussions with study team members with extensive experience working with Kenyan facilities. FGDs were led by formally trained and highly experienced Kenyan facilitators who had worked with study researchers in other projects. FGDs were conducted in a mix of English, Kiswahili, and/or Dholuo, lasted between 65 and 140 min, and were audio-recorded. FGD facilitators wrote debriefing reports after each interview, highlighting main observations and their own reflections on the group dynamics, including their interactions with the group. Interviewers transcribed audio recordings of the FGD they facilitated verbatim, and then translated them where necessary. Transcripts were quality checked by senior study team members. Both IDIs and FGDs explored perspectives and experiences of delivering PrEP as an integrated component of SRH services provided to AGYW. Participants in both IDIs and FGDs provided written informed consent.

### Data Analysis

Interview transcripts were uploaded to Atlas.ti version 8 (Scientific Software Development GmbH, Berlin, Germany). FGD transcripts were uploaded into Dedoose version 6.1.18 (SocioCultural Research Consultants, LLC, Los Angeles, California, USA). Codebooks for each project were developed through a combination of deductive and inductive methods. Deductive codes were generated from several implementation science frameworks (27–29). Inductive codes were then added through multiple reviews of the transcripts by qualitative analysts. Using a final codebook, interview transcripts were coded by one researcher (SR) while a second researcher (GO) reviewed coded transcripts and noted areas of disagreement. For FGD transcripts, three qualitative researchers (KB-S, AW, and GO'M) divided and independently coded transcripts which were then exchanged and reviewed by a secondary coder who noted areas of disagreement. Disagreements in coding for both IDIs and FGDs were resolved through discussion and consensus. Thematic content analysis (30) was conducted first for each data set (PrIYA FGDs and POWER IDIs) separately, and then common themes across both projects were compiled for this analysis.

## Results

We conducted 36 IDIs with HCWs engaged with the POWER study in six facilities in Kisumu, Cape Town and Johannesburg. We conducted eight FGDs with a total of 50 HCWs affiliated with the PrIYA study across 37 facilities in Kisumu. Most, though not all, of our participants were recruited and trained by the POWER and PrIYA studies. Demographic information about study participants is described in Tables 1, 2. FGD and IDI participants described an active ‘learning by doing’ approach as needed to successfully integrate delivery of PrEP into existing SRH services at their facilities. Participants anticipated HCW motivation as being the biggest challenge to sustained and scaled service integration. Common thematic challenges and strategies related to service integration and PrEP delivery to AGYW are described below. Additional illustrative quotations are included in Table A1.

**Table 1:** POWER key informant characteristics.

	**Cape town**	**Johannesburg**	**Kisumu**	**All sites**
**N participants interviewed**	11	10	15	36
**Age**	32 (27–43)	40 (36–43)	30 (29–42)	33 (29–42)
**Female**	7 (64%)	8 (80%)	9 (60%)	24 (67%)
**POWER-affiliated^a^**	10 (91%)	6 (60%)	10 (67%)	26 (72%)
**Primary occupational role** ^**b**^				
*Healthcare provider*	8 (73%)	6 (60%)	10 (67%)	24 (67%)
HCT counselor	3 (37.5%)	2 (33%)	3 (30%)	8 (33%)
Clinician^c^	3 (37.5%)	2 (33%)	6 (60%)	11 (46%)
Other	2 (25%)	2 (33%)	1 (10%)	5 (21%)
*Other key informant*	3 (27%)	4 (40%)	5 (33%)	12 (33%)
**Years working as healthcare provider** ^**d**^	8 (5–10)	10 (6–10)	6 (3–8)	6 (4–10)
**Years working in PrEP delivery**	2 (1–2)	3 (2–3)	2 (1–2)	2 (1–2)

a *Participant was considered POWER-affiliated if s/he currently or formerly worked for the POWER study*.

b *Based on participants primary role vis-à-vis PrEP and POWER. For example, a participant who is a doctor by profession but whose primary role in POWER is as a study coordinator, is counted as “other key informant.”*

c *Clinicians include nurses and doctors/medical officers*.

d *Excludes interviewees from the category “other key informant.”*

**Table 2:** PrIYA focus group characteristics.

	**Value**
Number of Focus Groups	8
Number of Focus Group Participants	50
Age in years: median (IQR)	28.0 (26.0, 32.0)
**Cadre**	
Peer counselor	3 (6%)
Clinical officer	8 (16%)
Nurse counselor	6 (12%)
Doctor	2 (4%)
Nurse	31 (62%)
Counselor	4 (8%)
Other	2 (4%)
Number of years at current clinic, median (IQR)	1.5 (1.0, 2.7)
Number of years delivering PrEP, median (IQR)	1.1 (0.8, 1.5)
PrIYA staff	27 (54%)

### Learning by Doing: Problem-Solving by Front-Line HCWs Aimed to Strengthen Provision of Patient Centered PrEP Services

HCWs described an ongoing process of active learning and modification of practices to integrate PrEP into existing reproductive health services within the context specific to each clinic.

…* [W]e, from different facilities, had to find something that would work in wherever we were working, because at the end of the day what will work for this facility might not work for the other, and we had to come up with ways to make PrEP delivery better for the future generation* (PrIYA, FGD 2, Participant 1).

HCWs noted that AGYW were extremely sensitive to perceived judgmental attitudes and stigma associated with sexual activity and PrEP, and hence were very concerned with their privacy. Once initial plans were made and PrEP delivery begun, HCWs reworked delivery processes aimed at improving patient centered care for their AGYW population.

For example, HCWs identified several approaches to minimize the time their young PrEP clients spent in public queues at the clinic, thus lessening their exposure to other clients at the facility whom AGYW feared might judge them. In many clinics HCWs implemented a practice of “fast tracking” PrEP clients by either moving PrEP clients to the head of the queue or by having a single clinician provide multiple aspects of PrEP services in the same consulting room, e.g., HIV testing, counseling, and prescribing.

…*[O]nce they come for their clinic day, they need to be fast tracked. … She just goes straight to the clinician, she is given a prescription, then straight to pharmacy. [She] doesn't want to be in the line. When you do like that, you will find them coming back* (PrIYA FGD 5, Participant 4).

In many facilities in Kenya, PrEP is dispensed from “ART pharmacies,” that is, pharmacies known to be dedicated to provision of antiretroviral therapy for individuals living with HIV. HCWs reported AGYW did not want to be seen queuing outside these pharmacies due to the stigma associated with being thought to be HIV-infected. In response, some nurses volunteered to pick up PrEP themselves from the ART pharmacy and then dispensed it from their clinic rooms.

HCWs also learned that AGYW often wanted to keep their PrEP use private and were concerned their family members or friends might see the PrEP bottle or hear the tablets rattling. To respond to this concern, some HCWs reported transferring the medication to small plastic bags for distribution so storage of a month's supply of tablets would be more discrete.

*Okay, we always repackage the PrEP into a zip lock bag, yeah, that one after we had a meeting of all the employees of the program and we agreed that even if you repackage it, it will not affect the efficacy. So, we always repackage it to a zip lock bag, and they like it that way* (PrIYA FGD 1, Participant 1).

Finally, although both South Africa and Kenya have formal PrEP risk assessment tools, HCWs explained that these formal risk assessments could make clients feel judged. In response, many HCW described fine-tuning their risk assessment and counseling techniques to support AGYW in making their decisions about whether they needed/wanted PrEP.

*You tell them, “Hey, I'm here for you. So, tell me about your partner.” So, once they start telling you about their life and their partner, you—both of you together—you can start to do assessments like, “So you said he comes late and he's drinking. It's okay. That is his life, and you are used to it. But, you see, there are certain risks.” So, you explore [PrEP] with them and ask, “Is it viable for you or not?”* (POWER, Kisumu IDI 3).

HCWs also reported adjusting their counseling advice to acknowledge the fluidity of AGYW sexual relationships, the multiple demands on their time and attention, and affirming their clients' autonomy in deciding whether or how long to use PrEP. Several health care providers used analogies between PrEP and family planning methods to convey key messages about duration of use.

*I found that comparing it [PrEP] to contraceptives, it works a lot, like, “You are doing contraceptives, and you can stop anytime you want when you feel that you no longer want to use them…. [Similarly, PrEP] will help you for as long as you need it…” It [the analogy] works well, and you realize that they understand it quickly when you go that route* (POWER, Johannesburg IDI 11).

### Motivational Challenges Due to PrEP Delivery Being Seen as Extra Work and Potential Solutions

Although HCWs in POWER and PrIYA experienced a learning curve in terms of incorporating PrEP into existing SRH services, they all described such service integration as feasible, as long as there was health care worker motivation. Lack of front-line staff motivation was highlighted by participants as the biggest anticipated challenge to scaling up PrEP. They predicted that since HCWs in public SRH clinics typically experience challengingly high patient volumes, they would consider PrEP provision as “added work.”

*If you want to add a service to an existing service, the first words you will hear are, “We're already working so hard. You want to add extra now for the same pay?” It's not going to happen. So that in itself is a barrier* (POWER, Cape Town IDI 10).

Despite the inclusion of oral PrEP in national HIV prevention strategic frameworks, many HCWs reported having had minimal training or exposure to PrEP, little awareness of why they were being asked to engage in PrEP service delivery, and uncertainty about PrEP's safety and efficacy.

*At first … I was like, “Why do you want to give people ARVs and they are not HIV-positive? …. It is going to lead to maybe drug resistance? …” At that point, I was not that enlightened about PrEP because I had known about HIV and ARVs and stuff, but very little about PrEP* (POWER, Kisumu IDI 17).

Study participants emphasized that in order for scaled delivery of PrEP as an integrated component of SRH to succeed, HCWs need to understand not only *what* PrEP is but *why* it is being brought in. Our study participants emphasized the important role of sensitization and training of those who are responsible for direct service provision.

*It's good to prepare staff. That is the first point for me, because when we arrived at [site name], people were raw in terms of “What are we doing? What is PrEP all about? Why is it necessary?” … If they understand how PrEP works, and you try to troubleshoot whatever fears or concerns they may have, it'll be easy [to scale PrEP up] if these other sites are convinced from the word ‘go’ of why PrEP has to be rolled out* (POWER, Johannesburg IDI 04).

Study participants reported that many of the HCWs they encountered were generally unaware that PrEP was part of national healthcare policy. Participants believed that if PrEP policies were disseminated more purposively to HCWs, they would be less inclined to view PrEP provision as extra work.

*[One potential challenge to scale-up] is if it's seen as extra work rather than standard of care. …. [P]eople see all the new programs—HIV and AIDS [services], PrEP, even AYFS [adolescent and youth-friendly services]—they see it as extra work. And [they think] that's not their work because they were employed to do this [other thing] … But if you have a memo from the National [Department of] Health, people will do whatever the memo says* (POWER, Johannesburg IDI 1).

They also emphasized the importance of the in-charge nurse or lead clinical officer being able to refer to national guidelines to normalize PrEP provision as an integrated component of SRH services.

“*[T]he nurse in-charge and the clinical officer, they made us realize that PrEP is in the guidelines. So, it's not that we're doing something new. It is something that's supposed to be in existence already running…. So that made us not feel like, “This is out of place” …* (POWER, Kisumu IDI 3).

Finally, several of our participants advised addressing short-term workload concerns by emphasizing the longer-term benefits of reducing HIV incidence in the population, and thus stemming increases in HIV client volume at their health facility.

*I think often the problem with something like PrEP … is that the benefits of them are long-term. And it's hard as someone in the thick of it who sees 60 patients a day to see that, by doing this now, you're … reducing your patient load [in the] long term* (POWER, IDI Johannesburg 10).

### Motivational Challenges Due to Social Stigma and Concerns Around Promiscuity

The most frequently referenced barrier to HCW motivation to deliver PrEP was their uneasiness with adolescent sexuality and their moral concerns regarding sexual activity among unmarried young women. Participants were often told by their colleagues that suggesting a young woman begin PrEP would be the same as encouraging her to have unprotected sex.

*I think most people, including some of the health workers, still think that when we talk about PrEP we are encouraging promiscuity or early sex* (PrIYA FGD, participant 1).

*[Initially,] there was a belief [among some health care workers] that this [PrEP] will cause young women to be more promiscuous … [and HCW were] not wanting to have those conversations with young women …* (POWER, Cape Town IDI 2).

IDI and FGD participants frequently described working in locations with “conservative” community and religious norms. Participants emphasized that HCWs are also part of these communities, and frequently share community moral concerns about young women's sexual relationships. These moral reservations in turn negatively impacted motivation and willingness to provide PrEP services.

*Actually, when it started, it was a slow move because people would argue, “Why give the medication? That one is promotion for promiscuity”* (POWER, Kisumu IDI 9).

*They [HCW] are still reluctant about it [providing PrEP]…[T]hey are thinking if you are making this PrEP available and so effective in preventing HIV, you are saying to these kids, to these women, to be promiscuous, that's some of their thinking* (POWER, Johannesburg IDI 11).

### Overcoming Moral Concerns and Reluctance to Provide PrEP

Study participants reported a variety of strategies to overcome HCW moral reservations about providing PrEP. One approach was to link HIV prevention to a woman's valued role in society, e.g., that of mother.

*[I told colleagues] “This PrEP is not for people to be promiscuous. It's for helping people. And even if someone is a kind of sex worker and she's using PrEP, it's still better for her because you know at least she'll stay HIV negative for her children”* (POWER, Kisumu IDI 3).

HCWs in our study also tried to overcome colleagues' moral reservations by prioritizing HCWs' professional obligation to keep clients healthy, regardless of their personal opinions about sexually active AGYW.

*What I would advise them [colleagues] is when trying to talk to the adolescent, “Try and push that client first. Irrespective of your opinion or the feelings you have, put the interests of the client first … so that you may be able to …help as a healthcare worker”* (PrIYA FGD 2, Participant 1).

Study participants also reported trying to persuade their colleagues of the value of providing PrEP through a pragmatic emphasis on the empirical realities of AGYW being sexually active, and their professional responsibility to keep them HIV-free.

*Now, you need to realize why did this adolescent come to the clinic for family planning, meaning this girl is going to have unprotected sex, meaning HIV here is not catered for. So, they [HCWs] need to understand that these people are having sex, and they need to take action, yes, despite their values, because at the end of the day they need to protect them. Yes, you will continue shouting, “Abstain! Abstain!” They are not abstaining* (PrIYA FGD 4, Participant 5).

In addition, our study participants tried to motivate colleagues to offer PrEP by highlighting the social reality of power differentials between young women and their male partners. In terms of HIV protection, this meant emphasizing PrEP as an empowerment option for women.

*PrEP is better when compared with other methods of HIV prevention because, you see, for PrEP, the woman or the lady herself controls it. You see, for a man, he might decide whether to wear condom or not, or when you tell a lady to abstain, the guy might decide whether to abstain or not. But you see, for PrEP, it is within her own control…* (PrIYA FGD 2, Participant 5).

*[PrEP] gave people more options and more room to make more informed decision and to really feel empowered… [you] don't depend on [your] partners' ‘go-ahead’….So I thought it was a really great option”* (POWER, Johannesburg IDI 06).

Some of the health providers interviewed in this study reported urging colleagues to empathize with their young clients' vulnerability. One participant described how encouraging co-workers to imagine members of their own family needing PrEP convinced them that delivering PrEP to AGYW was the right thing to do.

*Initially, there was the issue of staff attitude in [facility name], but you know, the good thing is that we got to explain to them that “It can also be your daughter [who needs PrEP] or it can also be you. Maybe today you are here, you know your partner's status, [but] next time, he may be HIV-positive, and you need PrEP.” … So, I think that is what made them feel like, “I am also a human being”* (POWER, Kisumu IDI 3).

Several of our participants described how their own initial moral reservations to providing PrEP to AGYW dissipated over time as they gained a greater appreciation of AGYW's HIV risk and began viewing PrEP as an opportunity to intervene.

*[Sometimes I'm] like, “Oh my goodness” … But now you think [to yourself], “This is a girl who is at risk. Okay, I have my own values. I have my own beliefs, you know, but now I have to help these young girls, because if I don't, maybe no one will”* (POWER, Kisumu IDI 8).

## Discussion

HCWs in Kenya and South Africa engaged in delivering PrEP in two studies across 43 facilities described iterative learning to integrate PrEP into SRH services for AGYW. Examples of ‘learning by doing’ included repackaging PrEP pills from bottles to plastic bags, modifying their counseling messages, and streamlining client flow in an effort to make integrated PrEP delivery with SRH services friendlier to adolescents. HCWs believed the biggest challenge to sustained integration and scaling of PrEP for AGYW would be health care worker motivation, primarily due to a tension between personal and professional values and expectations.

The need for iterative learning and practice modifications in order to integrate and scale new health services has been extensively described in the implementation science literature (31–39). Given significant variability in capacity and infrastructure across facilities where SRH services are delivered as well as in the communities they serve, there will not be a single “blue print” for integration of PrEP (40). Successfully integrating new services has been described as mirroring other complex adaptive systems, wherein actors (HCWs and other stakeholders) must continually reinvest energy over time to mobilize resources and engage in an ongoing process of adaptation to refine and realign clinical practices to make them workable, and to meet evolving stakeholder choices, concerns, and expectations (35, 39, 39, 41, 42). This ongoing investment of time and effort relies on frontline health workers being motivated to exercise agency in the adaption and implementation process.

**Figure 1:**
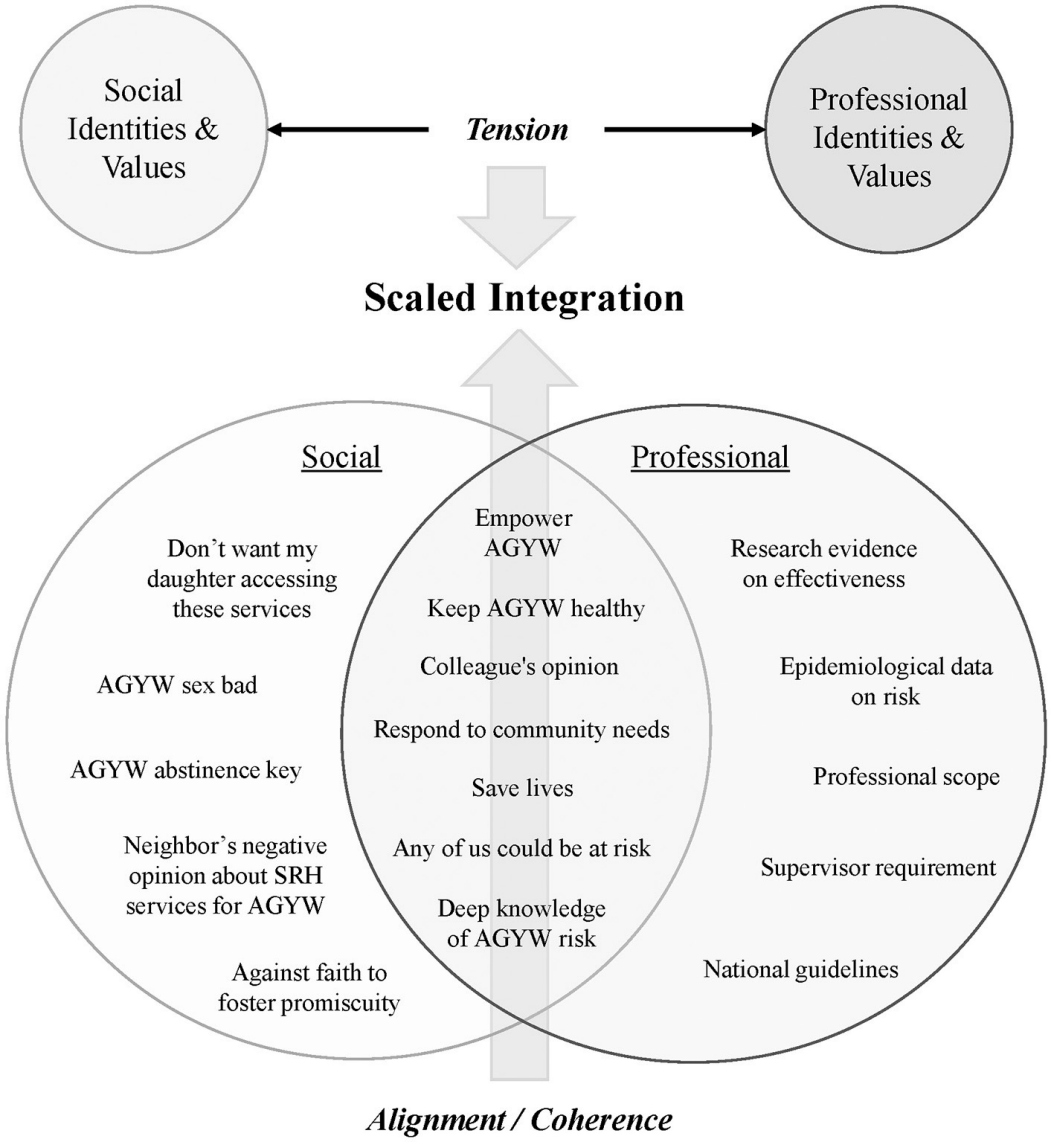
Health care worker motivation.

Within the context of these two implementation studies, IDI and FGD participants described their on-going efforts to make PrEP service integration workable and to meet client needs. However, they also predicted health worker motivation would be one of the most likely barriers to delivering PrEP at scale as an integrated component of SRH outside of study contexts. Process evaluations of the interaction of HIV testing, care, and treatment services have similarly identified motivation as a key factor influencing service integration (14, 20, 42). More broadly, health worker motivation has been identified as a prime factor influencing performance of an expected task (39, 41, 43–46). Capacity to provide a new service consists of both “*can do*” and “*will do*” components. Skills-based training, national guidelines, and basic materials (e.g., PrEP medication) facilitate the *can do* of service integration. However, intrinsic motivation is essential to whether providers *will* do so [(36, 47, 48); Figure 1].

Participants in our study highlighted moral reservations about providing PrEP to AGYW which negatively impacted HCW motivation to invest in the work necessary for service integration. The primary moral concern expressed was not wanting to foster sexual promiscuity. Other studies have similarly described HCW concerns with PrEP provision as condoning or encouraging sexual promiscuity among AGYW, MSM, transgender women, injection drug users, and Black Americans, resulting in lowered health provider willingness to prescribe PrEP to these clients (49–54). More generally, in a systematic review of health care worker motivation, the construct of “moral norms” was found to be a significant determinant of intention, and intention predictive of provider's behavior (55, 56).

HCWs belong to multiple communities in their personal and professional capacities which may have different values and expectations related to PrEP provision. Although national guidelines and formal trainings (professional community) may clearly articulate expectations supporting integration of PrEP with other SRH services for young women, HCWs are also heavily influenced by their social worlds (extended family, neighbors, and faith communities) which may hold very different norms, expectations, and values (38, 39, 43, 56–58). The lack of congruence or alignment between personal and professional expectations and values may add a psychological burden as health providers exert effort to resolve the tension between the two, serving as a demotivating influence on the task-focused work of PrEP and SRH integration (45, 59). While individuals can hold multiple beliefs about a behavior or an intervention, the psychological principle of *salience* suggests one can attend to a relatively small number of beliefs at any given moment (60, 61). Our study participants described several strategies of persuasion they used to help co-workers resolve this tension, either through encouraging alignment between the personal and professional or be encouraging the salience of professional values as they tasked with integrating PrEP and SRH for AGYW.

**Figure 2:**
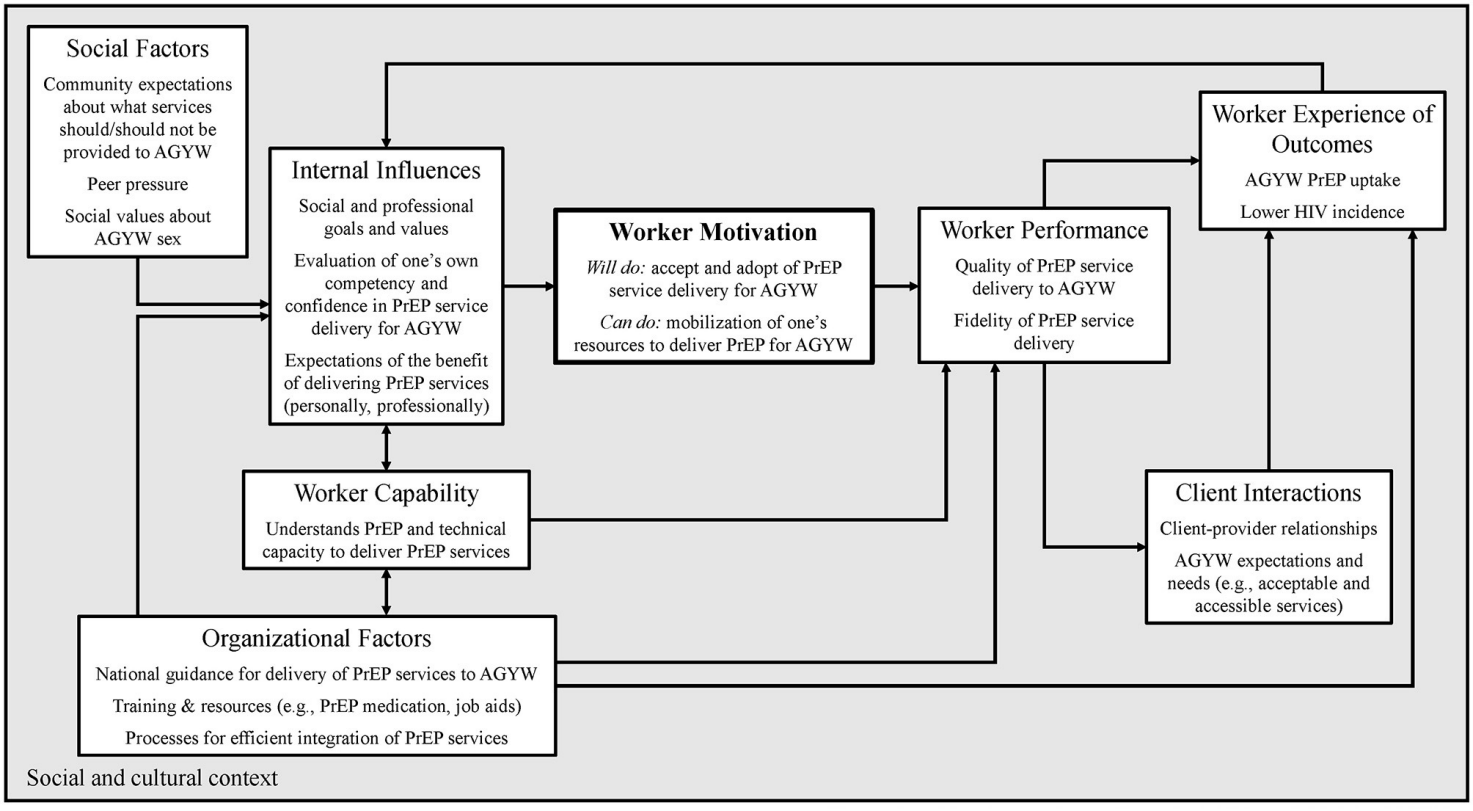
Health care worker motivation and values alignment. Figure adapted from Franco, Bennet, Kanfer, Social Science & Medicine 54 (2002).

First, they urged colleagues to prioritize their professional identities and the associated vocational responsibilities of keeping young women healthy. The construct of “professionalism,” i.e., how closely health care providers identify with the values and expectations associated with their profession, has been documented as an important motivator to implementing evidence-based practices (56, 62). Second, our study participants described entreating their colleagues to recognize AGYW's disproportionate HIV risk and societal norms which often constrain a young woman's ability to keep herself HIV-free, either via abstinence or the use of condoms, and highlighted the potential of PrEP to empower women to protect themselves. Health care worker belief that an intervention is well-placed to meet a client's specific needs has been identified as an important facilitating factor in intervention implementation (27). Finally, our study participants sought to strengthen their colleagues' motivation through appealing to their empathy, urging them to imagine they or their female relatives needed PrEP. The more closely HCWs can align their own perceived risks and needs with those of their client population and their community, the more highly motivated they may be to provide the intervention (41, 63, 64). All of these strategies may contribute to HCWs developing a sense of “coherence” around PrEP and SRH service integration for AGYW. Coherence has been described as a set of beliefs drawing from professional, social, and personal identities, which facilitate actionable meaning-making about an intervention [(45, 56, 62, 65); Figure 2]. The more frontline HCWs perceive a service or practice as meaningful and useful, the more highly motivated they will be and the more likely they are to expend the necessary energy for its implementation (41, 63).

Moral concerns around women's sexual activity are not unique to PrEP delivery (66, 67). HCW judgmental and censoring attitudes, especially toward sexual activity of adolescent and unmarried women, have been identified as discouraging the provision and use of contraceptive services by these populations (68–76). Although the WHO and national governments have called attention to the need for adolescent-friendly reproductive health services, progress in public clinics has been slow, though moving in the right direction (77). In spite of natural synergies across the provision of PrEP and other SRH services to AGYW, the success of such planned integration will be limited if moral reservations around family planning are amplified by similar reservations about PrEP among frontline health workers.

There is some evidence that formal training can facilitate changes in HCW attitudes to SRH services to be more client centered, although the outcomes vary greatly depending on training methodologies and content (78, 79). For example, using participatory methods and/or the inclusion of adolescent “standard patients” during training may facilitate shifts in provider attitudes (79–81). Targeted recruitment and use of AGYW as local “PrEP champions” or opinion leaders as trainers, colleagues, or supervisors, may also positively influence shifts in provider attitudes and subsequent practice (82–84). Although widespread positive attitudes about SRH for AGYW, including PrEP, will not in themselves be sufficient for scaled integration and implementation, these will be foundational to a facilitative context (76, 85).

Maximizing the synergistic potential of SRH and PrEP should also consider services integration *outside* of health facilities. In many countries, policy shifts and product innovations are enabling increased access to SRH services without increasing patient volume at SRH clinics. For example, pharmacies and drug shops are important sources for oral contraceptive pills, emergency contraceptives, and condoms (86). In recent years, a subcutaneous contraception injection product containing medroxyprogesterone acetate (DMPASC) has been developed and shown it can be safely used by both community health workers and via self-injection (87). Well-designed digital health applications providing educational information can be highly acceptable to clients for accessing reproductive health information (88). Innovations such as these should also be considered for PrEP as a means toward broadening access for AGYW (89).

An important limitation of our analysis is that it was conducted within the context of two studies, with more resources and training available than in purely programmatic settings. Both PrIYA and POWER hired health care providers as study staff to provide technical assistance and some direct support for integrated PrEP/SRH services. HCW descriptions of workload and motivational challenges could be different outside of a research context. However, all of our study participants had extensive experience with public health clinics in their countries, and not all of the providers interviewed in this project were directly employed by POWER and PrIYA. Motivational issues are likely to be even more important outside of a research context.

## Conclusion

While policies, guidelines, and facility-specific protocols are certainly essential tools to guide the integration of PrEP into other SRH services for AGYW, frontline health care workers must be motivated to implement them. HCWs individually and collectively must see the value of and find positive meaning in an intervention such as PrEP for it to become embedded into routine services and practice. Meaning is motivating, and motivation is crucial for the reflexive monitoring and feedback necessary to integrate, modify to evolving context, and hence scale interventions. Decades of family planning research have identified health care worker moral reservations or opposition to providing contraception to adolescents and young women as having a negative impact on the delivery and uptake of contraceptive services. As efforts move forward to integrate PrEP into family planning services for African AGYW, programs should anticipate and proactively work toward overcoming these same concerns.

## Data Availability Statement

The datasets presented in this article are not readily available because request for data sets will require approval of partners in South Africa and Kenya. Requests to access the datasets should be directed to Gabrielle O'Malley, gomalley@uw.edu.

## Ethics Statement

The studies involving human participants were reviewed and approved by Ethics committees at the University of Cape Town, the University of the Witwatersrand in Johannesburg, the Kenya Medical Research Institute (KEMRI), the Kenyatta National Hospital Ethics and Research Committee and the University of Washington Institutional Review Board. The patients/participants provided their written informed consent to participate in this study.

## Author Contributions

KB-S, SR, GB, and JD organized the database. GO'M, GB, SR, KB-S, and AW coded the data. GO'M wrote the first draft of the manuscript. All authors contributed to conception and design of the study, manuscript revision, read, and approved the submitted version.

## Conflict of Interest

The authors declare that the research was conducted in the absence of any commercial or financial relationships that could be construed as a potential conflict of interest.

## Appendix

**Table A1:** Additional illustrative quotations.

**Themes**	**Illustrative quotations**
Learning by doing—facility context	When we went to those facilities, what we had in mind changed because each facility is unique (PrIYA FGD 5, participant 4). Judging by our clinic flow currently and how it's working, I think sitting down and discussing how best we can also make it work for PrEP. So we will have to redesign some of the things because we go back and see is this working, is this working…. So how can we best accommodate them [PrEP clients] into the clinic that is already in existence (POWER Johanessburg, IDI 3).
Learning by doing—dispensing	Initially when we started, we would write the prescription, and then we let them go and pick the medication from the pharmacy. But being a facility that was seeing so many patients and [because] that pharmacy was the pharmacy where we also had people living with HIV [getting ARVs]…most of the time the queues would be long. And when you sent the young woman for PrEP there, they would, like, take more time. … The second [issue] is that the staff … in the pharmacy felt like by them dispensing PrEP, we were adding them more work, and they were not really comfortable to dispense the PrEP. And so we decided that we'd let the nurse or the clinician prescribe the medication and also dispense the medication….[W]e would keep the drugs in the pharmacy, but in the morning when we open the clinic, we would take enough for that day (POWER, Kisumu IDI 17).
Learning by doing—counseling on risk assessment	The feedback we were getting was that people were quite resistant to being told they were at risk. And that is something I can totally relate to. I would hate it if I went somewhere and someone told me I was at risk based on my behavior. Like, “Excuse you?”…. But I think that we spent a lot of time thinking about how to change that narrative…. And so I think we learned a lot about how to talk about risk (POWER, Johannesburg IDI 10).
Concerns with PrEP encouraging promiscuity	Because PrEP is associated with issues of sexuality, there is always this concern about how we are promoting promiscuity. “Are we encouraging people to have sex?” (POWER, Kisumu IDI 13). And to many people, they are thinking if you are making this PrEP available and so effective in preventing HIV, you are saying to these women to be promiscuous. … But we try by all means to advise or show them that … we are not saying people should be reckless because they are on PrEP (POWER, Johannesburg IDI 11).
Concerns with PrEP encouraging promiscuity negatively impacting service provision	[W]e find some clinics where have . staff who believe strictly that some things are meant to be done when people are adults or when people are in marriage and should [not] be done at some other time—so for this kind of staff, they will not be open to giving the young women whatever they need. If they see a young woman coming maybe for family planning, they will be like “Why are you here? You are supposed to be in school. You are supposed to doing something else.” And that discourages young women from interacting with them (POWER, Kisumu IDI 17). …[L]ike I have seen whereby a nurse…a female nurse, now feels like she is the mother to this girl and she is seeing this girl is now promiscuous, she becomes so annoying, shouts at her and when such a thing happens you find that the girl is scared and tends to stop opening up anymore (PrIYA, FGD 2 participant 5).
Conflicting personal and professional values and beliefs	To some of us health workers we tend to believe that sex should begin at a given age such that when we encounter maybe an adolescent who is 15 years of age in need of PrEP, we start doubting whether it is true or not, we tend to [project] our own beliefs on that client. So it is high time we need to change our attitude so that we get to know that some of the adolescents, actually they have early sexual activity, their sexual activeness starts very early (PrIYA, FGD 6 participant 2). Some staff have values, you know, “Maybe I'm not supposed to offer family planning because I'm a Catholic. Catholics are against this” (POWER, Kisumu IDI 8).
Fostering motivation: focusing on the longer term	I even say to them [providers], ‘You remember we are trying to curb the spread of HIV, and people you are seeing for ART, you know, the number would be less if we now had this prevention option of PrEP. So we are working toward the same goal (POWER, IDI Johannesburg 11).
Fostering motivation: professional expectations	I started going [to trainings] with the policies, so I could be like, ‘This is a policy. It's just not widely disseminated yet.’ … [Providers] often say they want to see the research, but that's not quite what they mean. They want to see the legitimacy. … This is approved by government. This is something that we're now providing that's been legitimately approved, like it's been regulated by SAPRAA [Southern African Pharmaceutical Regulatory Affairs Association]. …. There have been studies that shown us this, and it's been regulated and approved by government and regulatory bodies around the world (POWER, Johannesburg IDI 10). …. [I]t comes down to things like personal motivation…. I think there are other staff motivational issues that can be put into place even if you can't influence the actual salary change. So that even if they provide this additional service that they may feel is onerous and is taking up our time and is an additional responsibility for which I'm not paid, then it's looking at “what are you here for?” You're here to provide a service. Now what is that service? That service could range from PrEP, to contraception, to ARV treatment, to putting plaster of Paris on broken bones, to whatever else. So helping them appreciate what they are doing and then helping them recognize they're appreciated for what they are doing. So that they also feel that they are important and that what they are doing is contributing the overall goals of the country, of the region, in achieving better health (POWER, Kisumu IDI 13).
Fostering motivation through seeing value to the community	People would argue, “Why give the medication? That one is promotion for promiscuity.” But it reached certain levels where people were seeing the importance [of PrEP] because the clients themselves were coming for it… And when we saw them coming, we got to know, “Oh! So this thing is very important to the people.” It is not important to the providers themselves. Those who consume it are now coming more and more, and they were really in good numbers. So that is the time we realized this is very important. …. [W]hen we were in training, we were also worried…. “What will people perceive about the PrEP?” But we came to realize that people are in need of it fully (POWER, Kisumu IDI 9). I'm sure at some point for them [HCW], they felt quite helpless as a helping professional. You know, you're trying to bring about a positive change in this person and to decrease the amount of risk that they are faced with. But there's really not that much that you can do. But PrEP sort of gave them another gateway, you know, to help these patients to take PrEP (POWER Johnaessburg, IDI 6).
Fostering motivation through continuing medical education	At initial stages people were a bit skeptical [about providing PrEP] and I think it was mainly with relation to what …they thought, that this would lead to more maybe promiscuity and you know the reserved cultures that maybe the hospital is [in], the catchment area, so they thought maybe it would lead to promiscuity. But again after the sensitization and the CMEs, they discovered that that is not the case, yeah so they gained a better reception after they got the information, yeah (PrIYA, FGD 8, participant 1).
Fostering motivation through highlighting professional values	[E]ven if it is an adolescent and the rest of the age groups we should treat them as clients so we should not impose our values on the patients and then as health care workers we should think that this is a preventing measure so what if you don't give this patient the preventive measure then now the client turns to be positive so it is better to prevent than to treat yes (PrIYA, FGD 6, participant 1). …[I]t is up on us as the health workers who are at those various stations [FP/MCH clinics] because the reason as to why we are here is to give quality service to our clients and all of us want to help in reduction of HIV prevalence in our country isn't it? So it is up on us to change our attitude and maybe not to wait for support supervision [laughter] because you know we always know the right thing that we should be doing there yeah… So it is upon us to embrace the new intervention that has come and give good services to our clients (PrIYA FGD 06, participant 2). My advice to them [reluctant HCW]is that they should just call a spade a spade and because they are tasked with the duty of giving service to mankind, they should just talk about PrEP, talk about sex and talk about everything, not hiding any information from the young women so that they see the light and follow the light (PrIYA, FGD 1, participant 4).
Fostering motivation through emphasizing social realities and professional role	The thing I'm seeing is that sometimes it's difficult for them to negotiate condom use. It's also difficult for them to say “no” to sex because sometimes they are forced into it. So I thought it [PrEP] was a really good idea (POWER, Johannesburg IDI 05). The thing that had the nurses embrace [PrEP] is just because we were dealing with the same group of people, because there is no way a girl can come for family planning and you are offering PrEP at the same time and not talk to her about PrEP. That was not fair because this girl has come to see me, so a girl has come to seek family planning and also she wants to test for pregnancy …. So it is just unfair that I will go, provide her with the [family planning] methods and not talk to her [about PrEP]. ….if someone is coming for contraception, it means that they are sexually active. She does not want to get pregnant, but what about HIV? So it puts you in a situation where you really need to talk to her about the need for her to be protected against HIV, and protecting yourself against HIV, that is giving PrEP (POWER, Kisumu IDI 01). My [initial] concern was that it [giving PrEP to AGYW] was like we were promoting promiscuity, like we were giving them a room. But later I realized that it [risk behavior] is still there, despite the fact that we are denying them [PrEP] … They will not stop [change their risk behavior] because you think they should be stopping (POWER, Kisumu IDI 5). It's mostly, the decision of a partner more than their decision, to use it [condoms] or not to use it. Because, if the male partner doesn't feel like using it, that means that the condom won't be used even though sex is gonna be happening….So it was quite a nice thing to hear about, and then I strongly felt that it's a good thing for them to have power on their hands as well so that they can their own informed decisions…and be in charge of their sexuality or sexual life as well (POWER, Cape Town IDI 3).
Fostering motivation through empathy	Because if you have people that pull in a different direction, that makes things difficult for you. But once people [are] of one in mind, and one in, “this needs to be done”, then it can be done. Most of them [HCW in our studies] are living in the townships…. So they knew also the risks in the townships. For me coming in to the township, and my findings as in, so many girls testing positive and so many girls [are] coming for contraceptives, and so many stories we hear. That was for me a driving force. So it was really, we can actually save someone here, that was the thing, we can actually save someone. ….So the reality for me was also evident, I could see that this can make a difference into someone's life (POWER, Cape Town IDI 10). What I know about PrEP now has really changed my—is it attitude. The way I viewed PrEP before is not the way I view it now, because now I understand we don't want our young people to get infected because they are the future generation. HIV has been like “God, so what do we do?” So if something can be done to protect this young generation, to me it is a plus. … I am happy, and now I can be more involved because now I understand what it is all about, and I cannot be judgmental. You know, before I was like, “You are giving [PrEP to] which people?” But now [I feel] it can even help your own child (POWER, Kisumu IDI 04). … I have family members who are HIV positive, that also has an influence of me, making things easy for myself to actually deliver PrEP because, my goal is to have an HIV-free generation…. I always tell stories that are happening because I am part of the community. I've been in the township; I know exactly what is happening there. …I always reference from what's happening in the township, and then uhm I think also they can relate to that, and uhm, it kind of flows. …And nobody wants to have HIV so, people want anything that is going to prevent them from getting HIV. And for now we have PrEP and it works (POWER Cape Town IDI 6).
